# Complete Genome Sequence of Pseudomonas aeruginosa CMC-097, Isolated from a Ventilator-Associated Pneumonia Patient, Containing a Novel Carbapenem Resistance Class 1 Integron

**DOI:** 10.1128/MRA.00774-21

**Published:** 2021-09-02

**Authors:** Jayasimha Rao, Adenike Adenikinju, Thomas M. Kerkering, Dorothy C. Garner, Roderick V. Jensen

**Affiliations:** a Division of Infectious Disease, Virginia Tech Carilion School of Medicine, Roanoke, Virginia, USA; b Department of Biological Sciences, Virginia Tech, Blacksburg, Virginia, USA; c Internal Medicine, Division of Infectious Disease, Carilion Medical Center, Roanoke, Virginia, USA; d Center for Emerging, Zoonotic, and Arthropod-borne Pathogens, Virginia Tech, Blacksburg, Virginia, USA; University of Delaware

## Abstract

We report the complete genome of a clinical strain of Pseudomonas aeruginosa CMC-097, which was isolated from a ventilator-associated pneumonia patient with a chronic infection. Illumina sequence reads were assembled using Geneious to yield a 7,044,064-bp circular chromosome containing a carbapenem resistance integron, In*2020*.

## ANNOUNCEMENT

Chronic and multidrug-resistant (MDR) Pseudomonas aeruginosa is a threat to ventilator patients, with increased mortality rates of up to 30% in intensive care units (1–3). A World Health Organization (WHO) survey reported that carbapenem-resistant (CR) P. aeruginosa ranked as the second most critical priority bacterium among 20 antimicrobial-resistant bacterial species (4). In the United States, CR P. aeruginosa was reported in 2004 for an isolate containing the Verona integron encoding a carbapenemase (5) associated with mobile insertional sequence (IS) elements that play a major role in global dissemination (6–8).

A prospective study was approved by the Carilion Clinic institutional review board and conducted from 2010 to 2012 (9). In this study, P. aeruginosa CMC-097 was obtained from the Quest Diagnostics microbiology laboratory at Carilion Clinic. The strain was isolated from a tracheal aspirate specimen from a chronic ventilator-associated pneumonia patient and was confirmed by antimicrobial tests to be CR (10). The isolate was grown on a blood agar plate and transported to the Carilion basic science research laboratory; glycerol stocks were made and stored at −80°C.

A single colony of CMC-097 was grown in 25 ml lysogeny broth at 37°C at 200 rpm for 18 h. The cell pellet was used for genomic DNA isolation by Genomic-tip 20/G (9, 11). The genome was sequenced on the Illumina NextSeq platform at the Virginia Tech Genomics Resource Center with a library constructed using the Illumina TruSeq DNA preparation kit. Sequencing generated 72,739,130 paired-end (PE) reads of 150-bp length, which were assembled with the Geneious v11.0.4 *de novo* assembly algorithm with the default low sensitivity/fastest settings, which allow at most 10% base mismatches (9). No additional read quality filtering was necessary. This resulted in 77 contigs larger than 1,000 bp, with a maximum length of 597,066 bp and an *N*_50_ value of 214,826 bp. Then the Geneious algorithm map to reference was used to map the PE reads to these 77 contigs, with fine tuning set to iterate up to 10 times and custom sensitivity settings set to 0% mismatch and 0% gaps, to iteratively extend the ends of the 77 contigs until the ends overlapped and all of the gaps were closed into a circular genome (9). Synteny with highly similar Pseudomonas genomes (strains W60856 and PABCH45) was used to assist in joining contigs separated by repeated sequences of IS elements and rRNA operons. Finally, Geneious was used to map 96% of the PE reads to this complete genome with a uniform average coverage of 1,487×.

The complete assembly of CMC-097 resulted in a circular genome of 7,044,064 bp, with a G+C content of 66.4%. The NCBI Prokaryotic Genome Annotation Pipeline (PGAP) v5.0 (6, 7) identified 6,632 genes (IAU57_00005 to IAU57_33160), including 6,467 protein-coding genes, 82 RNA genes (65 tRNAs, 12 rRNAs, and 5 noncoding RNAs), 83 pseudogenes, and 1 CRISPR array.

**FIG 1:**
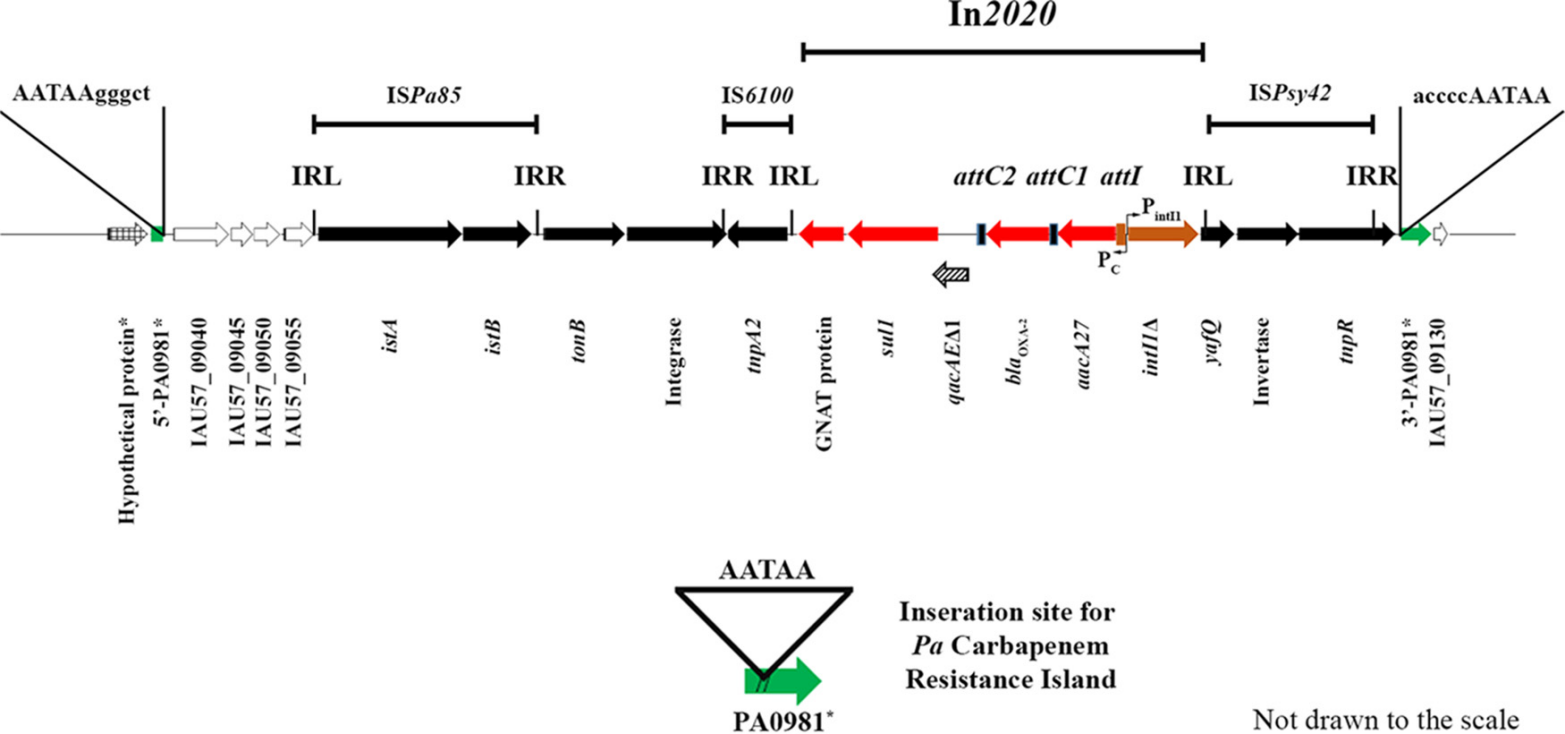
Antibiotic resistance island containing In*2020* in P. aeruginosa CMC-097 (nucleotide positions 1944662 to 1961187). The IS, inverted repeat, and transposon elements (*tnp*) were identified using ISfinder with their previously described names (13). The solid arrows represent the annotated open reading frames (ORFs) and their orientations, including the novel P. aeruginosa In*2020* containing the GCN5-related *N*-acetyltransferase (GNAT), *sul1*, *qacE*Δ1, *bla*_OXA-2_, and *accA27* resistance genes in red. Open arrows indicate ORFs for hypothetical proteins. The terminal direct repeats belonging to the insertion sites in the PA0981 gene are in green. The P_C_ (−10, nucleotide positions 1956769 to 1956774; −35, nucleotide positions 1956792 to 1956797) is the common promoter present in the integron, and P_intI1_ (−35, nucleotide positions 1956627 to 1956632; −10, nucleotide positions 1956650 to 1956655) is the promoter for the truncated integrase gene IntI1_R32_H39Δ_ (nucleotide positions 1956682 to 1957845). The recombination sites for IntI1Δ, *attI* (nucleotide positions 1956539 to 1956601), *attC1* (nucleotide positions 1955802 to 1955871), and *attC2* (nucleotide positions 19549326 to 1954995) are marked with vertical bars (12). Notably, insertion elements IS*Pa85* and IS*6100* were found upstream and downstream of *tonB* and another integrase. Also, downstream of In*2020* the IS*Psy42* element is inserted into the broken segment of PA0981. The inverted repeats at the 5′ and 3′ ends (left and right ends), i.e., the left inverted repeat (IRL) and the right inverted repeat (IRR), for the IS elements and transposons are marked.

BLAST analysis of the resulting genome found that it was highly similar to P. aeruginosa strain W60856 (GenBank accession number CP008864.2) and P. aeruginosa strain PABCH45 (GenBank accession number CP056101.1), with differences of <1/1,000 bp over genome stretches as long as 400,000 bp. However, the genome sequence was interrupted by many different IS elements, including 13 copies of an IS*21* family transposon, consisting of a transposase (*istA*) gene and a transposition helper (*istB*) gene (e.g., IAU57_01410 to IAU57_01415), and 9 copies of an IS*3* family transposase (e.g., IAU57_01325). P. aeruginosa CMC-097 also contains a CR class 1 integron, called In*2020*, which was defined and named by INTEGRALL (12) and is detailed in Table 1 and shown in Fig. 1.

**TABLE 1:** Genetic composition of the antibiotic resistance island containing the class 1 integron In*2020*

IS/integron element^*a*^	Gene identifier	Gene name	Direct/inverted repeat(s)^*b*^	Gene function^*c*^
		Hypothetical protein^*d*^		
		5′-PA0981^*d*^	DR: AATAAgggct	
	IAU57_09040	Hypothetical protein		
	IAU57_09045	Hypothetical protein		
	IAU57_09050	Hypothetical protein		
	IAU57_09055	Conserved protein		DNA recombination protein RmuC
IS*Pa85*	IAU57_09060	*istA*	IRL: tgcggattccacgctgactcggacacccattccacgcacatccgg	IS*21* family transposase
	IAU57_09065	*istB*	IRR: tgcggattccacgccattcggacactcagcccacgctgatccgga	IS*21*-like element ISUnCu3 family helper ATPase
	IAU57_09070	*tonB*		TonB C-terminal domain-containing protein
	IAU57_09075	Integrase		Tyrosine-type, site-specific recombinase/integrase
IS*6100*	IAU57_09080	*tnpA2*	IRL: ggctctgttgcaaagattggcggcagtcagagg; IRR: ggctctgttgcaaaaatcgtgaagcttgagcat	IS*6*-like element
In*2020*	IAU57_09085	*N*-Acetyltransferase		GNAT family protein
	IAU57_09090	*sul1*		Sulfonamide-resistant dihydropteroate synthase
	IAU57_09095	*qacE*Δ1		Quaternary ammonium compound efflux SMR (truncated) transporter
	IAU57_09100	*bla*_OXA-2_		Oxacillin-hydrolyzing class D β-lactamase
	IAU57_09105	*aacA27*		Aminoglycoside *N*-acetyltransferase AAC(6′)-IIc
	IAU57_09110	*intI1* ^*e*^		Class 1 integron integrase IntI1
IS*Psy42*	IAU57_09115	*yafQ*	IRR: aatgatgacctcaagccggttctggtcg	Type II toxin-antitoxin system
	IAU57_09120	Invertase		Recombinase family protein; DNA invertase Pin-like protein
	IAU57_09125	*tnpR*	IRL: aatgttctccgtggcccgcttccggccg	TnpR resolvase protein
		3′-PA0981^*d*^	DR: accccAATAA	
	IAU57_09130	Hypothetical protein		

a A novel integron was identified in this study.

b DR, direct repeat; IRL, left inverted repeat; IRR, right inverted repeat. Lowercase letters are used for gene sequences and capital letters are used for direct repeat sequences.

c Putative functions of the gene were identified from an NCBI protein BLAST search. GNAT, GCN5-related *N*-acetyltransferase; SMR, small multidrug resistance.

d Gene was not annotated in CMC-097.

e Gene was truncated and overlapped.

### Data availability.

The annotated complete genome assembly of Pseudomonas aeruginosa strain CMC-097 is available in GenBank under the accession numbers CP065848, SRR14783931, PRJNA660482, and SAMN15950776. The novel In*2020* sequence was registered at INTEGRALL, a web-based platform dedicated to integron identification, under the accession number CP065848 (http://integrall.bio.ua.pt/?acc=CP065848).

## References

[1] Williams BJ, Dehnbostel J, Blackwell TS. 2010. *Pseudomonas aeruginosa*: host defence in lung diseases. Respirology 15:1037–1056. doi:10.1111/j.1440-1843.2010.01819.x. 20723140

[2] Klompas M. 2019. Ventilator-associated events: what they are and what they are not. Respir Care 64:953–961. doi:10.4187/respcare.07059. 31346070

[3] Moore NM, Flaws ML. 2011. Introduction: *Pseudomonas aeruginosa*. Clin Lab Sci 24:41–42. doi:10.29074/ascls.24.1.41. 21404963

[4] Tacconelli E, Carrara E, Savoldi A, Harbarth S, Mendelson M, Monnet DL, Pulcini C, Kahlmeter G, Kluytmans J, Carmeli Y, Ouellette M, Outterson K, Patel J, Cavaleri M, Cox EM, Houchens CR, Grayson ML, Hansen P, Singh N, Theuretzbacher U, Magrini N, WHO Pathogens Priority List Working Group. 2018. Discovery, research, and development of new antibiotics: the WHO priority list of antibiotic-resistant bacteria and tuberculosis. Lancet Infect Dis 18:318–327. doi:10.1016/S1473-3099(17)30753-3. 29276051

[5] Toleman MA, Rolston K, Jones RN, Walsh TR. 2004. *bla*_VIM-7_, an evolutionarily distinct metallo-β-lactamase gene in a *Pseudomonas aeruginosa* isolate from the United States. Antimicrob Agents Chemother 48:329–332. doi:10.1128/AAC.48.1.329-332.2004. 14693560 PMC310168

[6] Hall RM, Collis CM, Kim MJ, Partridge SR, Recchia GD, Stokes HW. 1999. Mobile gene cassettes and integrons in evolution. Ann N Y Acad Sci 870:68–80. doi:10.1111/j.1749-6632.1999.tb08866.x. 10415474

[7] Mazel D. 2006. Integrons: agents of bacterial evolution. Nat Rev Microbiol 4:608–620. doi:10.1038/nrmicro1462. 16845431

[8] Deng Y, Bao X, Ji L, Chen L, Liu J, Miao J, Chen D, Bian H, Li Y, Yu G. 2015. Resistance integrons: class 1, 2 and 3 integrons. Ann Clin Microbiol Antimicrob 14:45. doi:10.1186/s12941-015-0100-6. 26487554 PMC4618277

[9] Adenikinju A, Jensen RV, Kerkering TM, Garner DC, Rao J. 2020. Complete genome sequence of *Pseudomonas aeruginosa* CMC-115, a clinical strain from an acute ventilator-associated pneumonia patient. Microbiol Resour Announc 9:e00595-20. doi:10.1128/MRA.00595-20. 32703835 PMC7378034

[10] Rao J, Adenikinju A, Gray JM, Colpitts L, Prorock A, Bao Y, Kerkering TM, Garner DC, Jensen RV. 2021. Comparative transcriptomic analysis of *Pseudomonas aeruginosa* isolates from ICU patients with acute and chronic pneumonia. Int Arch Med Microbiol 3:e012. doi:10.23937/2643-4008/1710012.

[11] Rao J, Susanti D, Childress JC, Mitkos MC, Brima JK, Baffoe-Bonnie AW, Pearce SN, Grgurich D, Fernandez-Cotarelo MJ, Kerkering TM, Mukhopadhyay B. 2018. Tn*2008*-driven carbapenem resistance in *Acinetobacter baumannii* isolates from a period of increased incidence of infections in a southwest Virginia hospital (USA). J Glob Antimicrob Resist 12:79–87. doi:10.1016/j.jgar.2017.08.017. 28899807

[12] Moura A, Soares M, Pereira C, Leitao N, Henriques I, Correia A. 2009. INTEGRALL: a database and search engine for integrons, integrases and gene cassettes. Bioinformatics 25:1096–1098. doi:10.1093/bioinformatics/btp105. 19228805

[13] Siguier P, Perochon J, Lestrade L, Mahillon J, Chandler M. 2006. ISfinder: the reference centre for bacterial insertion sequences. Nucleic Acids Res 34:D32–D36. doi:10.1093/nar/gkj014. 16381877 PMC1347377

